# Cario-Vascular System

**Published:** 1991-09

**Authors:** M. Papouchado


### Book Reviews

**CARDIOVASCULAR SYSTEM**
**(Clinical Film Viewing Series)**
**George G. Hartnell**
Clinical Press, 1990, 164 pp. £15.00
ISBN 1 85457 012 9

As Professor Rhys Davies notes in his foreword to this excellent book, the back bone of medical teaching, be it the ward round or the tutorial, is the illustrated case. In this book Dr. Hartnell, who has trained in cardiology as well as in radiology, serves up 80 of these. Each begins with a short history and a few (though not too many) details; there is an illustration, usually a plain chest film though others are included such as a ventriculogram, an arch aortogram, a CT scan or an echo. Images obtained using newer techniques such as digital subtraction angiography and magnetic resonance imaging are included. Then come the questions: What does this show? What is the significance? What further investigations would you perform to reach a diagnosis? Over the page comes the answer — and much more. The abnormal features on the illustration are explained as are their significance. If there is more than one possible diagnosis each is discussed and further investigations considered.

Thus, each case tests the readers knowledge, and then teaches him some more. This makes the book doubly useful though clearly, some knowledge of the topic is required. This is therefore not the book for the clinical medical student, though he may want to dip into it prior to finals to refresh his memory of what the plain chest film in aortic dissection, mitral valve disease or pulmonary oedama looks like. It is however for those studying for the MRCP or the FRCR, while those wanting (or needing) to test their own knowledge and to learn a few new facts will also find it useful.

The book is quite small, which imposes limitations as only 80 cases are presented. These are limited to diseases of the heart and great vessels. A few paediatric cases are included. X-rays are never easy to reproduce in books and articles, some of the finer detail being lost, but the quality of the illustrations in this book is on the whole quite good, for which the publishers and printers should be congratulated. The writing is clear and references have been kept to a minimum; what is needed is facts, and facts are given.

M. Papouchado

